# Residue Depletion and Withdrawal Interval Estimations of Sulfamonomethoxine or Doxycycline Residues in Chinese Taihe Black-Bone Silky Fowls

**DOI:** 10.3390/ani15050640

**Published:** 2025-02-22

**Authors:** Mengjun Ye, Lijuan Yuan, Qiegen Liao, Jianjun Xiang, Li Zhang, Qiushuang Ai, Suyan Qiu, Yifan Dong, Xiren Yu, Dawen Zhang

**Affiliations:** 1 Institute of Quality & Safety and Standards of Agricultural Products Research, Jiangxi Academy of Agricultural Sciences, Nanlian Road 602, Nanchang 330200, China; 13668043636@163.com (M.Y.); ylj198820062467@163.com (L.Y.); liaoqg2004@126.com (Q.L.); xjj101@sina.com (J.X.); zhangli9061@163.com (L.Z.); qai2539@163.com (Q.A.); qiusuyan@126.com (S.Q.); dongyifan0321@163.com (Y.D.); nkyyxr7012@163.com (X.Y.); 2 Key Laboratory for Quality and Safety Control of Poultry Products, Ministry of Agriculture and Rural Affairs of the People’s Republic of China, Nanlian Road 602, Nanchang 330200, China; 3 Laboratory of Quality & Safety Risk Assessment for Livestock and Poultry Products (Nanchang), Ministry of Agriculture and Rural Affairs of the People’s Republic of China, Nanlian Road 602, Nanchang 330200, China

**Keywords:** sulfamonomethoxine, doxycycline, Taihe black-bone silky fowl, residue, withdrawal time

## Abstract

Black-bone fowls are rich in melanin and regarded as being rich in nutritional value. However, the metabolism of antibiotics in black-bone fowls remains poorly understood. This study aimed to determine the tissue residue depletion kinetics of Sulfamonomethoxine (SMM) or Doxycycline in Taihe black-bone silky fowls. The tissue residue experiments involved a total of two hundred and forty Taihe black-boned silky fowls. The birds were raised to 100 days old in the same laboratory conditions and divided into a control group, SMM group, and Doxycycline group (80 birds for each group). The control group was provided normal drinking water. The SMM group was oral-administered SMM at concentrations of 50 mg/kg bw per day, and the Doxycycline group was oral-administered Doxycycline at concentrations of 40 mg/kg bw for 5 days, respectively. After the last dose, liver, kidney, muscle, and skin/fat were collected at various time points (0.16, 1, 3, 5, 7, 9, 12, 20, 30, and 40 days, eight birds for each time point). Concentrations of SMM or Doxycycline were analyzed using the UPLC–MS/MS method. The terminal elimination half-lives of SMM in muscle, skin/fat, liver, and kidney were 1.82 ± 1.24, 15.3 ± 4.97, 4.36 ± 1.49, and 5.42 ± 3.46 days, respectively, and 8.62 ± 2.82, 6.88 ± 2.06, 4.12 ± 1.28, and 1.53 ± 0.28 days for Doxycycline. The estimated withdrawal times (WDIs) of SMM were 4, 27, 10, and 12 days, while the estimated WDIs of Doxycycline were 18, 15, 4, and 6 days for muscle, skin/fat, liver, and kidney, respectively. The SMM or Doxycycline bioaccumulate in the skin/fat and may cause antimicrobial residues to be absorbed by human when the skin/fat is used in the diet. Doxycycline persists in muscle for a longer duration compared to SMM. This highlights the significance of muscle and skin/fat as a target organ for future food safety research.

## Simple Summary

Black-bone fowls are rich in melanin and regarded as being rich in nutritional value. However, the metabolism of antibiotics in black-bone fowls remains poorly understood. This study aimed to determine the tissue residue depletion kinetics of Sulfamonomethoxine (SMM) or Doxycycline in Taihe black-bone silky fowls. The tissue residue experiments involved a total of two hundred and forty Taihe black-boned silky fowls. Estimated withdrawal times (WDIs) of SMM were 4, 27, 10 and 12 days, while estimated WDIs of Doxycycline were 18, 15, 4 and 6 days for muscle, skin/fat, liver and kidney, respectively. The SMM or Doxycycline bioaccumulate in the skin/fat and may cause antimicrobial residues to be absorbed by human when the skin/fat is used in the diet. Doxycycline persists in muscle for a longer duration compared to SMM. This highlights the significance of muscle and skin/fat as a target organ for future food safety research.

## 1. Introduction

Poultry meat holds significant importance in the consumption of Chinese residents as it constitutes a principal protein source, is cost-effective, and gains wide acceptance among various groups. In 2023, China’s poultry meat production reached 25.63 million tons, and the use of antibiotics is essential to the poultry industry [1]. However, drug-residue-containing tissues can bring health risks to consumers, such as enhanced drug resistance, allergic responses, and potential direct toxic impacts [2].

Sulfamonomethoxine (SMM) is a kind of sulfonamides, and it effectively treats many kinds of diseases like Salmonella disease and Escherichia coli disease in fowls. SMM can prevent bacteria from using PABA to synthesize necessary folic acid and inhibit bacterial growth [3,4,5]. Once the amount of SMM in animal food exceeds the limit, it will seriously threaten human health. In order to control the use of sulfonamides, China, the European Union and other countries and organizations have made provisions on their maximum residue limits. In 2019, the Ministry of Agriculture and Rural Affairs of China promulgated the National Food Safety Standard Maximum Residue Limits for Veterinary Drugs in Food [6], and the requirement for sulfonamides is a maximum residue limit of 100 μg/kg for edible tissue in all food animals. Doxycycline is one of the most frequently used antibiotics in poultry farming [7], mainly used in the treatment of poultry diseases such as mycoplasma infection [8]. Doxycycline exhibits outstanding efficacy against both Gram-positive and Gram-negative aerobic as well as anaerobic pathogens [9]. Its oral absorption is fast and nearly complete, and it has an extended terminal elimination half-life. The tissue-penetrating ability of Doxycycline is remarkable; residue concentrations have been detected in the major organs and tissues, including the kidney [10]. After absorption and metabolism by poultry, these drugs remain in different tissue parts, and the maximum residue limits of different parts are also different. Several studies have investigated SA or Doxycycline pharmacokinetics, bioavailability, and toxicity, but few studies have investigated the effects of SMM or Doxycycline on the residue depletion profiles of edible tissues in chicken [11,12]. The frequent application of SMM or Doxycycline does not indicate that associated risks in edible animals have been thoroughly investigated. According to the Quality of Veterinary Medicines (the Ministry of Agriculture and Rural Affairs of China), the concentration of SMM in drinking water is 250 mg/L~500 mg/L, and the concentration of Doxycycline is 300 mg/L. Additionally, the allowable daily intake (ADI) of SMM is 0–50 μg/kg body weight, while the ADI of Doxycycline is 0–3 μg/kg body weight [6]. Therefore, according to the conversion of chicken drinking water requirements, we set the feeding concentration of SMM at 50 mg/kg body weight or Doxycycline at 40 mg/kg body weight per day.

Taihe black-bone silky fowls is a traditional local breed in China and a typical representative of black-boned chickens. In 2023, the number of Taihe black-bone chickens raised to the market weight reached 8.5 million. The growth cycle of purebred Taihe black-bone chickens should be at least 180 days, and its melanin content is higher than other poultry [13]. Some studies have shown that melanin in the body binds to drugs, extending its half-life and thus increasing its metabolic time [14]; the drug metabolism time and withdrawal times (WDIs) in Taihe black-bone silky fowls may be different from other poultry. Since oral administration is a common administration route for SMM or Doxycycline in poultry, it is important to determine the tissue-residue-depletion kinetic profiles of SMM or Doxycycline in poultry following oral administration.

In this study, the kinetic characteristics of residual tissue consumption in Taihe black-bone silky fowls after the oral administration of SMM or Doxycycline were first characterized. Then, according to the collected tissue residual consumption data, the tissue WDIs of SMM or Doxycycline in black-bone chickens was estimated by WT 1.4 software. Therefore, the purpose of this study was to provide a theoretical basis for the formulation of special veterinary drug use and withdrawal procedures for Taihe black-bone silky fowls by presenting a scientific WDI estimate.

## 2. Materials and Methods

### 2.1. Animals and Sample Collection

#### 2.1.1. Animals

The tissue residue experiment involved a total of two-hundred-and-forty Taihe black-boned silky fowls (Jiangxi Wang Beitu Co., Ltd., Ji’an city, China). Birds were vaccinated against Marek’s disease and confirmed without Salmonella and other pathogenic infections. Then, birds were raised to 100 days old (120 females and 120 males, 0.76 ± 0.1 kg) in the same laboratory conditions without other history of medication. They were determined to be healthy based on physical examination and divided into control group, SMM group, and Doxycycline group (80 birds for each group). The control group was provided normal water. The SMM group was oral-administered SMM (Guangdong Dahuanong Animal Health Products Co., Ltd., Yunfu, China) at concentrations of 50 mg/kg body weight per day for 5 consecutive days. Doxycycline group was oral-administered Doxycycline (Guangdong Dahuanong Animal Health Products Co., Ltd., Yunfu, China) at concentrations of 40 mg/kg body weight per day for 5 consecutive days, respectively. The SMM or Doxycycline was incorporated into the drinking water, and the concentration of SMM is 375 mg/L, while the concentration of Doxycycline is 300 mg/L. Animals were raised without other antibiotic and under uniform indoor conditions (with a 9 h light and 15 h dark cycle, temperature was 25 ± 5 °C, and humidity was 50–60%). The density of chickens in the cage is 20 per square meter and commercial feed (Tongwei Co., LTD, Chengdu, China) were provided ad libitum [2,15].

The protocol approved by the Experimental Animal Ethics Committee of the Jiangxi Academy of Agricultural Sciences and conformed to standards set forth by the Ministry of Agriculture of China. The required number of animals per sampling point referred to the guideline VICH GL48(R), which specifies that a minimum of six birds are required per sampling point for poultry depletion studies [16].

#### 2.1.2. Tissue Collection

At each time point (0.16, 1, 3, 5, 7, 9, 12, 20, 30, and 40 days after the last dose), 4 female and 4 male Taihe black-boned silky fowl of each group were randomly selected and euthanized by cervical dislocation [17]. The pectoralis major muscle, skin/fat, liver, and kidney were collected at various time points, and the birds were euthanized, cleaned in ice-cold PBS (pH 7.36) twice, frozen in liquid nitrogen, and stored at −80 °C [18].

### 2.2. Analysis of SMM or Doxycycline

Muscle, liver, kidney, and skin/fat samples were minced and homogenized in a homogenizer for 2 min. Five grams for each homogenized sample was weighed and placed into a 50-milliliter polypropylene centrifuge tube. Then, 10 mL of acetonitrile was added into each tube and shaken for 20 min. The mixture was shaken for 10 min with centrifugation performed at 2800× *g* [19].

Supernatants were transferred to a 100-mL polypropylene centrifuge tube. After that, 5 mL of monobasic potassium phosphate buffer and 8 mL of acetonitrile were added to the remaining tissue pellet and shaken for 20 min. The mixture solution was centrifuged at 2800× *g* for 10 min. The supernatants were combined, and 40 mL of water was added and centrifuged under the same conditions as before. The supernatant was directly loaded onto a solid-phase extraction cartridge that had been pre-treated by rinsing with 10 mL of water and 10 mL of acetonitrile.

SMM or Doxycycline was eluted from the column using 2.5 mL of a 0.1 mol/L ammonium acetate/methanol/acetonitrile solution. The collected eluate was evaporated to dryness and then reconstituted in 1 milliliter of the mobile phase. The solution was filtered through a 0.2-micrometer syringe filter and analyzed by ultra-high-performance liquid chromatography-mass spectrometry (UPLC–MS/MS, Agilent Technologies, Inc., Agilent 1290 Infinity UPLC equipped with a 6460 Triple-Quadrupole mass spectrometer, Santa Clara, CA, USA).

### 2.3. Method Validation

The stability of the UPLC–MS/MS system and the feasibility of the determination method for Sulfamonomethoxine or Doxycycline was validated on muscle, liver, kidney, and skin/fat of Taihe black-bone silky fowls at 0.1, 1, and 10 μg/mL levels. Quality control samples (0.1, 1, and 10 μg/mL) were prepared in control matrix with each analysis along with a matrix blank. Control matrices were collected from muscle, liver, kidney, and skin/fat from non-medicated Taihe black-bone silky fowls. The recoveries were carried out on muscle, liver, kidney, and skin/fat, and the inter-day and intra-day precisions were obtained by extracting and analyzing five replicates (from different individuals) of fortified samples under same conditions. The standard curve of Sulfamonomethoxine was Y = 370.4X − 194.7 (range: 1.0–50.0 ng/mL), and the linear correlation coefficient was 0.9990. The within-day recoveries were 68.1–90.3% in muscle, liver, kidney, and skin/fat. The within-day coefficients of variation (CVs) ranged from 5.1 to 8.8% for samples. The between-day recoveries were 63.5–91.7%, with CVs of 6.3–10.1%. The standard curve of Doxycycline was Y = 1558.2X + 399.4 (range: 2.0–100.0 ng/mL), and the linear correlation coefficient was 0.9997. The within-day recoveries were 64.6–87.9% in muscle, liver, kidney, and skin/fat. The within-day CVs ranged from 5.9 to 8.3%. The between-day recoveries were 60.9–86.3%, with CVs of 6.0–9.8%. The limit of detection (LOD) and limit of quantitation (LOQ) for Sulfamonomethoxine and Doxycycline were 2 μg/kg and 10 μg/kg, respectively [20].

### 2.4. Pharmacokinetic Analysis [21]

The pharmacokinetic parameters of SMM or Doxycycline were evaluated using the time-dependent concentration data derived from tissue samples. A non-compartmental analysis (NCA) was conducted with the help of a commercial software program (Phoenix WinNonLin 6.1, Certara, Princeton, NJ, USA). The following pharmacokinetic parameters were automatically computed by the software program, and their definitions are presented below.

Cmax: The highest concentration of SMM or Doxycycline observed in tissues.

Tmax: The time at which the maximum concentration of SMM or Doxycycline is observed in tissues.

λz: The estimated elimination rate constant.

T_1/2_: The terminal elimination half-life, which is calculated by the ratio of the natural logarithm of 2 to λz.

AUC_0−∞_: The area under the concentration–time curve in tissues from the dosing time extrapolated to infinity based on the last observed concentration.

AUC extrapolation: The percentage of AUC_0−∞_ extrapolated from Tlast to infinity.

AUMC_0−∞_, Area Under the Moment Curve from time of dosing extrapolated to infinity.

MRT_0−∞_: The mean residence time (MRT) extrapolated to infinity.

Concentration–time plots for the tissue samples were created using a commercial graphing software program (GraphPad Prism 8.1, GraphPad Software, La Jolla, CA, USA).

### 2.5. Estimation of Withdrawal Intervals

In this study, the WT 1.4 software (EMA, European) was employed for the calculation of WDIs. Data regarding concentrations beneath LOD were excluded from the WDI calculation process. The WDI was estimated as the period during which the 95th percentile tolerance limit of the residue concentration was equal to or lower than the tolerance/LOD with a 95% confidence level.

## 3. Results

### 3.1. Residue Depletion Profiles in Muscle, Skin/Fat, Liver, and Kidney Tissues

**Figure 1:**
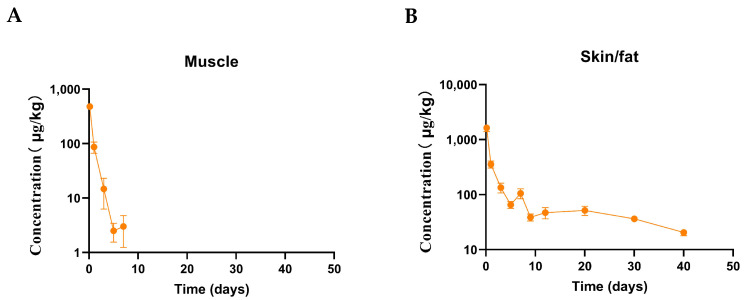

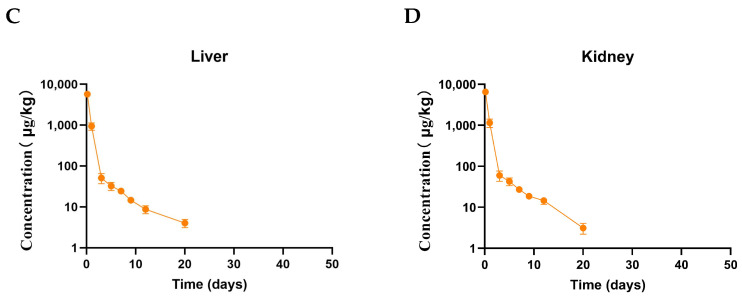
The mean concentration (mean ± SEM)–time curves for SMM following oral administration at 50 mg/kg bw/day for five days in Taihe black-bone silky fowls (at 0.16, 1, 3, 5, 9, 12, 20, 30, and 40 days after the last dose, n = 8 for each time point), the Y axis using a semi-logarithmic scale. (**A**) Muscle, (**B**) skin/fat, (**C**) liver, and (**D**) kidney.

The mean concentration–time curves of SMM in edible tissues are presented in Figure 1, and the drug elimination time was shortest for muscle and longest for skin/fat. The muscle, skin/fat, liver, and kidney pharmacokinetic parameters were calculated using a NCA approach, and the data are summarized in Table 1. Following the oral administration of 50 mg/kg of SMM for five days, compared with other tissues, the kidney had the highest Cmax, and muscle had the lowest Cmax. The terminal elimination half-lives for SMM were shortest in muscle (1.82 ± 1.24 d) and longest in skin/fat (15.30 ± 4.97 d). The AUC extrapolations of edible tissues were skin/fat > muscle > kidney > liver and less than 20%. The MRT_0−∞_ of edible tissues were skin/fat > muscle > kidney > liver. The results showed that there was a potential residual accumulation risk of SMM in the skin/fat of Taihe black-bone silk chicken if the withdrawal period was not followed.

**Figure 2:**
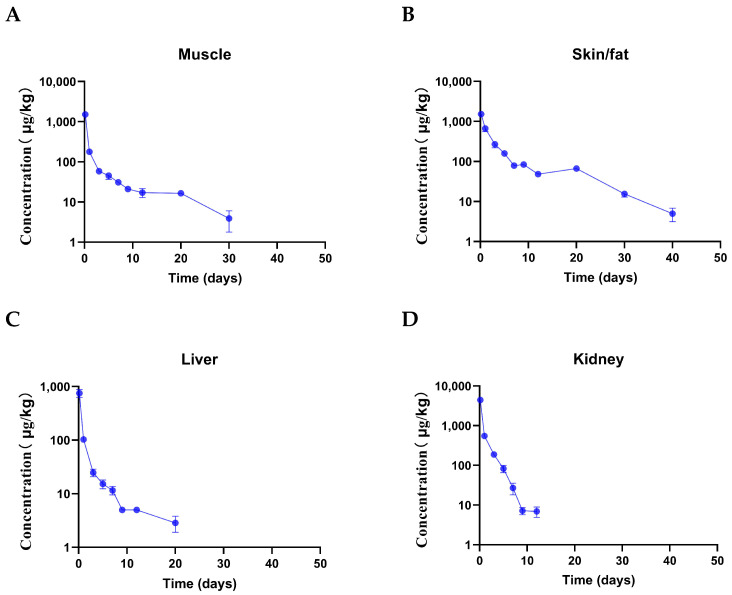
The mean concentration (mean ± SEM)–time curves for Doxycycline following oral administration at 40 mg/kg bw/day for five days in Taihe black-bone silky fowls (at 0.16, 1, 3, 5, 9, 12, 20, 30, and 40 days after the last dose, n = 8 for each time point), the Y axis using a semi-logarithmic scale. (**A**) Muscle, (**B**) skin/fat, (**C**) liver, and (**D**) kidney.

The mean concentration–time of Doxycycline in edible tissues are presented in Figure 2, and the drug elimination time for kidney and liver was shorter than muscle and skin/fat. For Doxycycline, the kidney had the highest Cmax, and liver had the lowest Cmax. The terminal elimination half-lives for Doxycycline were shortest in kidney (1.53 ± 0.28 d) and longest in muscle (8.62 ± 2.82 d). The AUC extrapolations of edible tissues were muscle > liver > skin/fat > kidney and less than 20%. The MRT0−∞ of edible tissues was skin/fat > muscle > liver > kidney (Table 2). The results showed that there was a potential residual accumulation risk of Doxycycline in the muscle of Taihe black-bone silk chicken if the withdrawal period was not followed.

### 3.2. Estimations of Withdrawal Intervals

**Figure 3:**
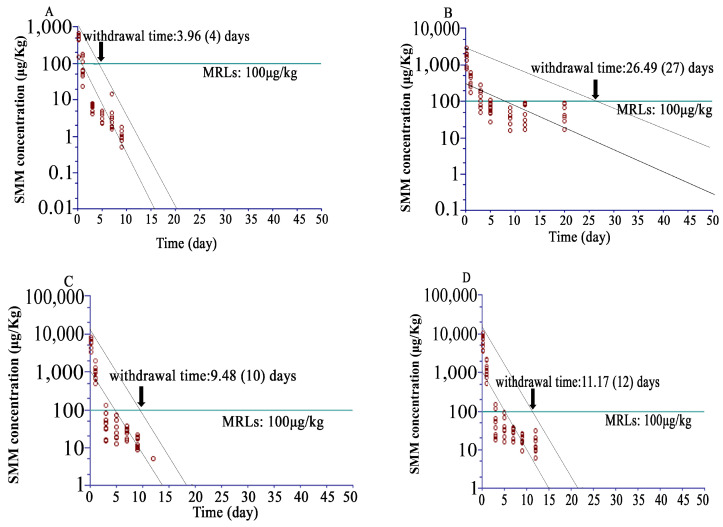
Concentration–time profile across different time points for SMM following oral administration at 50 mg/kg bw/day for five days in Taihe black-bone silky fowls (at 0.16, 1, 3, 5, 9, 12, 20, 30, and 40 days after the last dose, n = 8 for each time point). The horizontal line at 100 μg/kg represents the maximum residue limit (MRL), the dotted lines, the upper and lower 95% CI. (**A**) Muscle, (**B**) skin/fat, (**C**) liver, and (**D**) kidney.

The WT 1.4 software can only analyze the dataset with maximally seven time points. Thus, for SMM, the time points of 0.16, 1, 3, 5, 7, and 9 days were selected to calculate the withdrawal time for muscle and 0.16, 1, 3, 5, 9, 12, and 20 days for kidney. Time points 0.16, 1, 3, 5, 7, 9, and 12 days for skin/fat and kidney were selected to calculate the withdrawal with 95% confidence interval and maximum residue limits (MRLs) of 100 μg/kg for these issues. At all time points, SMM concentrations in edible tissues were above LOD (2 μg/kg), and the estimated WDIs were 4, 27, 10, and 12 days for muscle, skin/fat, liver, and kidney, respectively (Figure 3).

**Figure 4:**
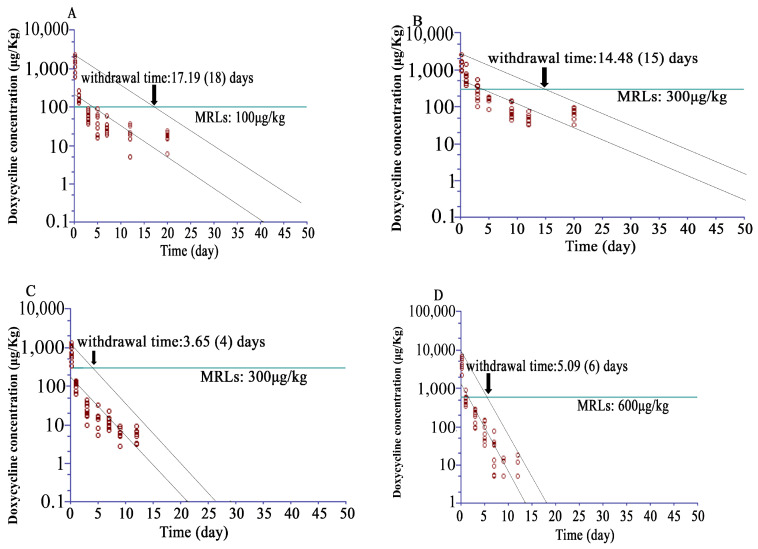
Concentration–time profile across different time points for Doxycycline following oral administration at 40 mg/kg bw/day for five days in Taihe black-bone silky fowls (at 0.16, 1, 3, 5, 9, 12, 20, 30, and 40 days after the last dose, n = 8 for each time point). Dotted lines, upper and lower 95% CI. (**A**) Muscle, the horizontal line at maximum residue limit (MRL) of 100 μg/kg. (**B**) Skin/fat, the horizontal line at MRLs of 300 μg/kg. (**C**) Liver, the horizontal line at MRL of 300 μg/kg. (**D**) Kidney, the horizontal line at MRL of 600 μg/kg.

For Doxycycline, the time points of 0.16, 1, 3, 5, 7, 12, and 20 days were selected to calculate the withdrawal time for muscle and 0.16, 1, 3, 5, 9, 12, and 20 days for skin. Time points 0.16, 1, 3, 5, 7, 9, and 12 days for liver and kidney were selected to calculate the withdrawal with 95% confidence interval. At all time points, Doxycycline concentrations in tissues were above the LOD (2 μg/kg); the MRLs of Doxycycline of 100 μg/kg for muscle, 300 μg/kg for skin/fat or liver, and 600 μg/kg for kidney were set. The estimated WDIs were 18, 15, 4, and 6 days for muscle, skin/fat, liver, and kidney, respectively (Figure 4).

## 4. Discussion

The growth cycle of Taihe black-bone chickens takes 180 days; therefore, it is necessary to select 100-day-old birds as research objects. In addition to our previous research, the recommended withdrawal time of tilmicosin for this breed after oral administration for 3 consecutive days at a dose of 75 mg/L in water should be 32 days [22] so the collected samples at various time points (0.16, 1, 3, 5, 7, 9, 12, 20, 30, and 40 days) are necessary. This study aims to provide basic information into the metabolism and accumulation in Taihe black-bone silky fowl tissues for estimating WDIs that assure food safety.

This study reports tissue pharmacokinetics and residue depletion profiles and estimates the WDIs of SMM and Doxycycline in Taihe black-bone silky fowls following an oral administration of SMM at 50 mg/kg and Doxycycline at 40 mg/kg, once a day for 5 consecutive days. The main results of this study included three points. First, the terminal elimination half-lives of SMM were 15.3, 5.42, 4.36, and 1.82 d for skin/fat, kidney, liver, and muscle, respectively; and the terminal elimination half-lives of Doxycycline were 8.26, 6.88, 4.12, and 1.53 d for muscle, skin/fat, liver, and kidney, respectively. Second, compared with other tissues, skin/fat had a high SMM and Doxycycline residue concentration and a long-estimated WDIs, which highlights the importance of skin/fat as a target organ for future food safety research. Third, muscle had the highest Doxycycline residue concentration and the longest estimated WDI, and muscle had the lowest SMM residue concentration and the shortest estimated WDI, which highlights the importance of providing a safe withdrawal to meet the regulatory compliance, especially when the fowl meat is imported from or exported to a different country.

In poultry, there are few studies to characterize the residual time of SMM in muscle tissue, and the residual time of Doxycycline in muscle is longer than that in fat [23,24]. In our study, kidney had the highest average concentration of SMM, followed by liver, skin/fat, and muscle. In a study for laying hens, a common biological half-life of SMM in the kidney, liver, muscle, and adipose tissue was estimated to be 5.2 h, and the disappearance of SMM from the tissues was rapid [25]. The Cmax of SMM is consistent with the results of other species; in tilapia, the Cmax of SMM in the order of magnitude was kidney > liver > muscle [26]. The MRT of SMM in the order of magnitude was skin/fat > muscle > kidney > liver but that of Doxycycline was skin/fat > muscle > liver > kidney. Short MRT values means high clearance [27]; the results indicated that SMM and Doxycycline accumulated mainly in the skin/fat. SMM may be attributed to its marked absorption from the skin/fat of Taihe black-bone silky fowls due to its higher lipophilicity. A study found that SMM has a higher partition coefficient value, which means the higher the lipid solubility [11]. A study has showed Doxycycline residues simultaneously in muscle, fat, and liver tissues after the administration of the broiler chicken, and residues in fat persisted for a shorter time than in muscle [28]. In contrast, our results showed that Doxycycline residues in skin/fat persisted for a longer time than in muscle.

According to the MRLs of SMM (100 μg/kg in muscle, skin/fat, kidney, and liver) or Doxycycline (100 μg/kg in muscle, 300 μg/kg in skin/fat and liver, and 600 μg/kg in kidney) set in China, the calculated withdrawal times of SMM were 4 days in muscle, 10 days in liver, 12 days in kidney, and 27 days in skin/fat and those of Doxycycline were 4 days in liver, 6 days in kidney, 15 days in skin/fat, and 18 days in muscle. Hence, the recommended withdrawal time of SMM for Taihe black-bone silky fowls after oral administration for 5 consecutive days at a dose of 50 mg/kg should be more than 27 days and that of Doxycycline for Taihe black-bone silky fowls after oral administration for 5 consecutive days at a dose of 40 mg/kg should be more than 18 days. Tissue residue depletion profiles play a vital role in estimating tissue WDIs, and the poultry WDIs of SMM and Doxycycline are 28 days (Announcement of the Ministry of Agriculture of the People’s Republic of China, No. 278). The government establishes antibiotic WDIs in edible tissues to ensure the safety of the poultry productions. Commonly, higher amounts of drugs are distributed in organs like the liver and kidney [29]. If specific tissues incorporate residues at higher concentrations, these tissues should be selected for residue monitoring [30]. The results indicate a high accumulation of SMM and Doxycycline in skin/fat, and Doxycycline has a long withdrawal period in muscle. These may be caused by the large amount of melanin in skin and muscle as the melanin binding of drugs is known to increase drug concentrations and retention in pigmented tissues [31]. These results may be helpful to regulatory agencies as they determine what tissues are to be monitored to ensure that the established residue safety tolerance levels are not exceeded.

## 5. Conclusions

The consumption analysis of SMM and Doxycycline described in this study suggests that these drugs bioaccumulate in the skin/fat and may cause antimicrobial residues to be absorbed by humans when the skin/fat is used in the diet. Doxycycline persists in muscle for a longer duration (18 d) compared to SMM (4 d). Therefore, conducting monitoring and control over fowl meat that has been treated with this antibiotic is essential for safeguarding consumer health and enhancing public health. These results may be helpful to regulatory agencies as they determine what tissues are to be monitored to ensure that the established residue safety tolerance levels are not exceeded. Additionally, this highlights the importance of suitable withdrawal times to ensure the quality of broiler products, especially when the fowl meat is imported from or exported to a different country.

## Figures and Tables

**Table 1:** Pharmacokinetic parameters estimated from SMM concentrations in tissue samples collected from Taihe black-bone silky fowls.

Parameter (SMM)	Muscle	Skin/Fat	Liver	Kidney
Cmax (μg/kg)	479.96 ± 155.49	1635.73 ± 662.70	5727.16 ± 1861.54	6538.25 ± 2512.54
Tmax (d)	0.16	0.16	0.16	0.16
T_1/2_ (d)	1.82 ± 1.24	15.30 ± 4.97	4.36 ± 1.49	5.42 ± 3.46
λz (1/d)	0.51 ± 0.26	0.05 ± 0.02	0.18 ± 0.08	0.17 ± 0.09
AUC_0−∞_ (d*μg/kg)	452.23 ± 74.08	3695.48 ± 615.92	4549.92 ± 1011.40	5336.29 ± 1358.02
AUC extrapolation (%)	5.48 ± 7.64	12.34 ± 5.63	0.74 ± 0.26	0.99 ± 0.62
AUMC_0−∞_ (d*μg/kg)	537.24 ± 428.12	63,548.07 ± 28,064.25	4795.91 ± 1436.31	6045.00 ± 1844.49
MRT_0−∞_ (d)	1.22 ± 1.05	16.66 ± 4.38	1.06 ± 0.23	1.16 ± 0.36

Note: The values represent mean ± SD; Cmax, maximum observed concentration in tissues; Tmax, time of maximum observed concentration in tissues; T_1_/_2_, terminal elimination half-life; λz, the estimated elimination rate constant; AUC_0−∞_, area under the concentration–time curve in tissues from time of dosing extrapolated to infinity and * is used instead of a multiplication sign; AUC extrapolation, percentage of AUC_0−∞_ extrapolated from Tlast to infinity; AUMC_0−∞_, Area Under the Moment Curve from time of dosing extrapolated to infinity; MRT_0−∞_, mean residence time extrapolated to infinity.

**Table 2:** Pharmacokinetic parameters estimated from Doxycycline concentrations in tissue samples collected from Taihe black-bone silky fowls.

Parameter (Doxycycline)	Muscle	Skin/Fat	Liver	Kidney
Cmax (μg/kg)	1517.26 ± 659.77	1509.34 ± 515.60	752.96 ± 348.86	4445.43 ± 1607.32
Tmax (d)	0.16	0.27 ± 0.30	0.16	0.16
T_1/2_ (d)	8.62 ± 2.82	6.88 ± 2.06	4.12 ± 1.28	1.53 ± 0.28
λz (1/d)	0.09 ± 0.03	0.11 ± 0.03	0.19 ± 0.06	0.47 ± 0.12
AUC_0−∞_ (d*μg/kg)	1697.32 ± 438.56	3977.30 ± 834.42	696.31 ± 183.33	3642.44 ± 934.94
AUC extrapolation (%)	10.40 ± 5.05	2.11 ± 1.91	4.49 ± 1.63	0.47 ± 0.30
AUMC_0−∞_(d*μg/kg)	11,569.88 ± 3312.21	32,298.91 ± 7736.59	1891.29 ± 715.59	3947.08 ± 1430.12
MRT_0−∞_ (d)	7.31 ± 3.20	8.19 ± 1.32	2.78 ± 1.06	1.07 ± 0.21

Note: The values represent mean ± SD; Cmax, maximum observed concentration in tissues; Tmax, time of maximum observed concentration in tissues; T_1/2_, terminal elimination half-life; λz, the estimated elimination rate constant; AUC_0−∞_, area under the concentration–time curve in tissues from time of dosing extrapolated to infinity and * is used instead of a multiplication sign; AUC extrapolation, percentage of AUC_0−∞_ extrapolated from Tlast to infinity; AUMC_0−∞_, Area Under the Moment Curve from time of dosing extrapolated to infinity; MRT_0−∞_, mean residence time extrapolated to infinity.

## Data Availability

The original contributions presented in this study are included in the article. Further inquiries can be directed to the corresponding author.

## Abbreviations

The following abbreviations are used in this manuscript:

SMM	Sulfamonomethoxine
WDIs	Withdrawal times
UPLC–MS/MS	Ultra-high-performance liquid chromatography–mass spectrometry
CVs	Coefficients of variation
LOD	Limit of detection
LOQ	Limit of quantitation
NCA	Non-compartmental analysis
MRT	Mean residence time
AUC	The area under the concentration–time curve in tissues
AUMC	Area Under the Moment Curve
